# Computational instantaneous wave‐free ratio (IFR) for patient‐specific coronary artery stenoses using 1D network models

**DOI:** 10.1002/cnm.3255

**Published:** 2019-12-23

**Authors:** Jason M. Carson, Carl Roobottom, Robin Alcock, Perumal Nithiarasu

**Affiliations:** ^1^ Zienkiewicz Centre for Computational Engineering, College of Engineering Swansea University Swansea UK; ^2^ Data Science Building, Swansea University Medical School Swansea University Swansea UK; ^3^ Derriford Hospital and Peninsula Medical School Plymouth Hospitals NHS Trust Plymouth UK; ^4^ HDR UK Wales and Northern Ireland Health Data Research UK London UK

**Keywords:** coronary arteries, FFR, haemodynamic modelling, iFR

## Abstract

In this work, we estimate the diagnostic threshold of the instantaneous wave‐free ratio (iFR) through the use of a one‐dimensional haemodynamic framework. To this end, we first compared the computed fractional flow reserve (cFFR) predicted from a 1D computational framework with invasive clinical measurements. The framework shows excellent promise and utilises minimal patient data from a cohort of 52 patients with a total of 66 stenoses. The diagnostic accuracy of the cFFR model was 75.76*%*, with a sensitivity of 71.43*%*, a specificity of 77.78*%*, a positive predictive value of 60*%*, and a negative predictive value of 85.37*%*. The validated model was then used to estimate the diagnostic threshold of iFR. The model determined a quadratic relationship between cFFR and the ciFR. The iFR diagnostic threshold was determined to be 0.8910 from a receiver operating characteristic curve that is in the range of 0.89 to 0.9 that is normally reported in clinical studies.

## Graphical abstract

In this work we estimate the diagnostic threshold of iFR through the use of a one‐dimentional haemodynamic model. The 1D model is first validated through a comparison with invasive fractional flow reserve measurements, for which a diagnostic accuracy of 75.76% was achieved. The one‐dimentional model was then utilised to compare cFFR and ciFR on patient‐specific arterial geometries that were extracted via CCTA. The diagnostic threshold of iFR was determined to be 0.891 which is in the normal range reported from invasive clinical studies.

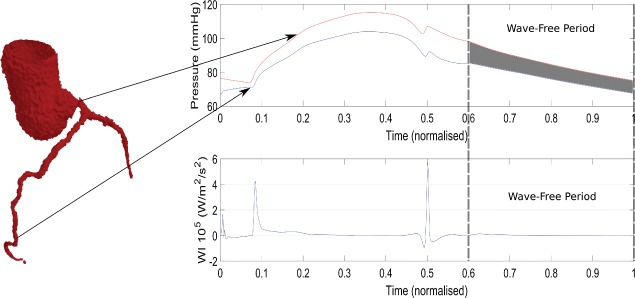

## 1 INTRODUCTION

Coronary heart disease (CHD) is the leading cause of mortality worldwide and has been attributed to approximately 15.5*%* of deaths per year. The prevalence of CHD creates a significant economic burden because of the direct health care costs, reduced productivity, and informal public aftercare caused by CHD and other cardiovascular diseases (CVD).^1^, ^2^ The risk factors of CHD and other types of CVD have been extensively studied and indicate the risk of developing a CVD is increased for individuals who smoke and those who are overweight.^3^ There have been efforts from health care systems aimed at encouraging populations to lead a healthier lifestyle^4^ as this has been shown to be effective at preventing many types of CVD and other diseases. However, even with this guidance, the prevalence of CHD (and other types of CVD) has continued to either remain constant or increase, with a striking finding that approximately 48*%* of all Americans have a CVD.^5^ Because of advances in medicine with improved diagnostic and treatment tools, the mortality rates caused by CHD are reducing in high‐income countries; however, this is not the case in middle‐income and low‐income countries where the number of deaths are increasing.^6^, ^7^ The economic burden is also expected to significantly increase as health projections have indicated there will be an increase in CHD and other types of CVD in part caused by ageing populations.^8^, ^9^ Thus, it is important to not only improve access to medical facilities that includes early diagnostic and treatment methods but also reduce the costs and increase the efficiency of the various patient treatment pathways for more sustainable health care systems.^10^

CHD is caused by a narrowing (stenosis) of a coronary artery and is often from the process of atherosclerosis. The current gold standard of care for assessing the functional significance of a stenotic lesion is fractional flow reserve (FFR).^11^, ^12^, ^13^ An FFR‐guided treatment strategy has been shown to reduce unnecessary stenting, reduce costs, and increase patient outcomes^14^, ^15^, ^16^, ^17^ compared with a strategy that utilises only quantitative coronary angiography. FFR is an invasive procedure that is performed during coronary angiography. The procedure requires a catheter and guide wire to be inserted into either the femoral artery or radial artery. The catheter is then guided to the ascending aorta where the coronary arteries are located and a pressure‐sensitive wire is then used to measure the pressure ratio from a point distal of the stenosis to a point proximal to the stenosis (aorta). Critical to the technique of FFR is that the measurement is performed under hyperaemic conditions and so requires a hyperaemic‐inducing drug to be administered to the patient. Hyperaemia increases the heart rate and cardiac output of the patient, which increases blood flow through the coronary arteries significantly.^18^, ^19^ However, a study showed that 28*%* of patients experience adverse reactions to adenosine, with 8*%* having such a severe reaction that the adenosine infusion had to be discontinued during the procedure.^20^ Other serious reactions to adenosine have also been reported, such as bronchospasms,^21^ tachyarrhythmia, and even cardiac arrest,^22^ although adverse effects as serious as these are rare.

Alternative diagnostic tools for CHD are continually being developed. One such technique is diastolic FFR (dFFR),^23^ which refers to a collection of variants to FFR. In the same way as the conventional measurement, dFFR requires a hyperaemic drug to be administered, and the main idea behind this variant is that the largest flow rates and lowest resistances in the coronary arteries are observed during diastole. The following are the main variants of dFFR: full dFFR, where the mean FFR over the entire diastolic phase is used; mid‐dFFR, where the FFR is measured at a single point in time (the middle of diastole); and end‐dFFR, where the FFR measurement is recorded only at end‐diastole by utilising an electrocardiography gating technique. End‐dFFR appears to be the most promising of these and has been shown to have a better correlation than conventional FFR to the FFR measured using flow probes,^24^ although few studies on dFFR currently exist.

Another promising alternative to FFR is the instantaneous wave‐free ratio (iFR).^25^, ^26^ The iFR procedure also requires a pressure sensitive catheter to be inserted in order to measure the pressure drop across stenotic lesion, but iFR is performed under resting conditions, so it does not require a hyperaemic‐inducing drug to be administered. The use of iFR was initially met with significant resistance, particularly by the community who strongly supported the use of conventional FFR^27^, ^28^ under maximum hyperaemia, but many studies have shown the viability and potential of iFR in diagnosing functionally significant stenotic lesions, showing similar diagnostic accuracy to conventional FFR^29^, ^30^, ^31^, ^32^, ^33^, ^34^, ^35^, ^36^, ^37^ and even showing a better repeatability than conventional FFR^38^ and a stronger correlation with the coronary velocity flow reserve.^30^ Questions still remain on whether iFR can reliably be used to replace FFR as several studies have shown mismatches in patient categorisation between iFR and the clinically trusted technique of conventional FFR,^32^ while the reasons for these disagreements were not fully understood. However, it has since been shown that the differences between the two indices was more likely related to FFR overestimating the severity of the stenosis, rather than iFR underestimating the severity.^39^ A hybrid iFR‐FFR approach has also been proposed,^38^, ^40^ which enhances the diagnostic accuracy while reducing the number of patients who require adenosine to be administered. In the DEFINE‐FLAIR study,^41^ iFR was shown to have noninferior patient outcomes when compared with conventional FFR and is considered a practical alternative to FFR that can avoid adverse patient reactions to hyperaemic drugs while costing less. The clinical cut‐off points of FFR and iFR are generally 0.8 and 0.89 respectively, although different cut‐off points have been proposed for iFR, ranging from 0.83 to 0.92,^29^, ^36^, ^42^ implying that the true cut‐off point for iFR is still uncertain. In the FFR‐iFR hybrid approach, a lesion with an iFR value in the grey‐zone of 0.86 to 0.93 also undergoes FFR, while an iFR value of less than 0.86 requires treatment and lesions with iFR values above 0.93 do not undergo surgical treatment.^38^, ^40^

Non‐invasive methods for estimating FFR are based on reconstructed and segmented coronary computed tomography angiography (CCTA) images. Techniques from computational fluid dynamics are then applied to the patient geometry to determine the FFR while avoiding the need for any invasive cardiac catheterisation. The technique is often called computed FFR (cFFR) and relies on the estimation of several important modelling components, which includes the patients' cardiovascular response to the hyperaemic drug infusion, which influences the elasticity of arteries, and boundary conditions of the model. Different computational methodologies have been proposed, which include computationally expensive three‐dimensional models,^43^, ^44^, ^45^, ^46^, ^47^, ^48^, ^49^, ^50^ and reduced‐order models.^51^, ^52^, ^53^, ^54^, ^55^, ^56^, ^57^, ^58^, ^59^, ^60^ One‐dimensional models have been shown to have excellent agreement with three‐dimensional models^53^, ^60^ but can be computed in seconds, rather than hours (for 3D). Computational models of iFR have been proposed but are generally compared with only invasive FFR measurements,^61^ in which the estimated iFR cut‐off point was 0.82; however, rigid‐wall conditions were assumed, which is known to significantly overestimate the pressure drop across a stenosis for cFFR^62^ and is likely to have a similar impact for ciFR or utilised in a hybrid‐approach,^63^ although a Monte Carlo simulation that utilises a lumped model^64^ without patient‐specific geometry has been implemented. Nevertheless, a comparison between the invasive iFR measurement and computational iFR was performed in Calmac et al.^65^ However, the computational methodology is not described in Calmac et al,^65^ requires images from invasive coronary angiogragraphy, and the cut‐off point for iFR is assumed to be 0.89.

The purpose of this work is to compare clinical FFR measurements with cFFR estimated via a one‐dimensional haemodynamic model in order to validate the cFFR strategy and to determine the diagnostic cut‐off point of iFR through the application of computational models on patient‐specific coronary arterial networks that have been extracted from non‐invasive CCTA. By using the same extracted patient‐specific coronary network geometry, the boundary conditions of the 1D model can be easily adjusted to change from hyperaemic conditions to resting physiological conditions, which essentially adapts the cFFR model into a ciFR model. The correlation between ciFR and cFFR and the clinically accepted FFR diagnostic threshold of 0.8 will then be used in order to estimate the diagnostic threshold for the iFR procedure. An advantage of using this computational framework is that variability of surgical techniques and of the patients' physiological conditions can be eliminated, which allows the iFR diagnostic threshold to be estimated in a more controlled environment. The patient data were collected retrospectively, and no patient specific measurements were used to aid the model parameter estimation.

The paper is organised into the following parts: in Section 2, the characteristics of the data utilised in this paper and the one‐dimensional computational framework are described; the results in Section 3 begin by comparing the cFFR predicted by the computational framework with the clinically invasive FFR measurements; this leads on to the comparison of the model predicted FFR and iFR values; in Section 4, the results are discussed and compared with previous findings in literature; and finally, the main conclusions of the work are presented in Section 5.

## 2 MATERIALS AND METHODS

### 2.1 Study characteristics

In this study, an anonymised retrospective data set of 52 patients with a total of 66 lesions was collected. The exact clinical FFR values were not available for a subset of 10 of these patients (15 stenosis). Only whether the FFR was positive or negative for these patients was available. An overview of the locations and characteristics of these stenotic lesions can be seen in Table 1. The majority of lesions (78.43*%*) considered in this work are in the intermediate FFR range of 0.7<*FFR*<0.9, with 68.2*%* of the total lesions having a negative FFR value and 31.8*%* having a positive FFR value. A stenosis location of the left anterior descending (LAD) artery was most prevalent (60.6*%*). Patients who had undergone previous coronary surgical interventions were included only if the intervention was performed within vessels that were not of interest for this study. Patients with serial stenoses and CCTA data images that contained motion artefacts and significant levels of calcification were also included in this study. Table 2 shows statistics on the characteristics of the CCTA images, which includes information on the percentage of cases with calcification and motion artefacts that are present.

**Table 1:** Distribution of stenosis location among the cohort and characteristics of the FFR measurements

Stenosis Location (n = 66)	n, %
LCA	3 (4.5)
LAD	40 (60.6)
LCX	7 (10.6)
DA	3 (4.5)
MA	2 (3.0)
IA	1 (1.5)
RCA	10 (15.2)

FFR Characteristics (n = 66)	n, %
FFR <0.8	21 (31.8)
FFR ≥0.8	45 (68.2)

Abbreviations: FFR, fractional flow reserve; LAD, left anterior descending.

**Table 2:** CCTA and stenosis characteristics

Stenosis No. and Type	Single Focal	Single Diffuse	Multiple
	50	16.67	33.33
Stent no.	0	1	2
	87.88	10.61	1.52
Occulsion no.	0	1	2
	83.33	15.15	1.52
Bifurcation	No	Within 1‐2 mm	Within 1 mm
	50	30	20
Calcification	None	Minor	Major
	22.73	48.48	28.79
Motion artefacts	None	Minor	Major
	74.24	19.7	6.06

*Note*. Percentage of cases includes the following: single focal stenosis; single diffuse stenosis; or if there are multiple stenoses (either focal of diffuse), number of stents and vessel occlusions, number of cases with a stenosis near (between 2 and 5 mm), and at (within 2 mm) of a bifurcation; whether calcification is present, and to what severity (minor is less likely to affect segmentation accuracy, while major is likely to impact segmentation accuracy); and whether motion artefacts are present and to what severity. All values given as a percentage of the cohort.

Abbreviation: CCTA, coronary computed tomography angiography.

### 2.2 Computed FFR methodology

The segmentation of CCTA images, centreline and vessel geometry extraction, were all performed in the image segmentation software VMTKLab, (Orobix, Italy).

#### 2.2.1 One‐dimensional haemodynamic model

The modelling methodology implemented in this work is described in Carson^66^ and involves the one‐dimensional haemodynamic equations in a pressure‐volumetric flow rate formulation^66^, ^67^ that is given by the conservation of mass:

(1) $$C_{a}\frac{\partial P}{\partial t}+\frac{\partial Q}{\partial x}=0,$$

where *C*_*a*_ is the vessel compliance, *P* is the mean hydrostatic pressure in a cross section, and *Q* is the volumetric flow rate; and the momentum equation:

(2) $$\frac{\rho }{A}\frac{\partial Q}{\partial t}+\frac{\rho }{A}\frac{\partial \left(\frac{Q^{2}}{A}\right)}{\partial x}+\frac{\partial P}{\partial x}=\frac{22\mu \pi Q}{A^{2}},$$

where *ρ*=1.05g/cm^3^ is the density of blood, *A* is the cross‐sectional area of the vessel, *μ*=0.04Poise is the blood viscosity, and the magnitude of the viscous coefficient of 22 is from Smith et al^68^ and was shown to be the best fit to experimental data for the coronary system. The vessel compliance is calculated as $\frac{\partial A}{\partial P}$ from the following viscoelastic constitutive law,^69^

(3) $$P-P_{0}-P_{ext}=\frac{2\rho c_{0}^{2}}{b}\left({\left(\frac{A}{A_{0}}\right)}^{b/2}-1\right)+\frac{\Gamma }{A_{0}\sqrt{A}}\frac{\partial A}{\partial P}\frac{\partial P}{\partial t},$$

with

(4) $$b=\frac{2\rho c_{0}^{2}}{P_{0}-P_{collapse}},$$

where the reference wave speed *c*_0_ is calculated from the vessel diameter using the empirical formula^69^, ^70^ as

(5) $$\frac{2}{3\rho }\left[k_{1}exp\left(k_{2}D_{0}/2\right)+k_{3}\right],$$

where *D*_0_ is the reference diameter, and the fitting parameters are *k*_1_=20 g/s^2^/cm, *k*_2_=−22.5 cm^−1^, and *k*_3_=86.5 g/s^2^/cm. The reference pressure *P*_0_ is set to equal the diastolic pressure, and the collapsing pressure *P*_*collapse*_=−10 mmHg. The wall viscous coefficient Γ^69^ is calculated from the following:

(6) $$\Gamma =\frac{100}{D}+400,$$

where *D* is the vessel lumen diameter.

The system of equations are solved using a subdomain collocation scheme^66^, ^71^ that is second‐order accurate in both time and space.

#### 2.2.2 Boundary conditions

The inlet and outlet boundary conditions for the model are predicted by using a two‐tiered parameter estimation technique. The first tier utilises a general, nonpatient‐specific, closed‐loop 1D‐0D network to estimate the volumetric inflow rate for the coronary arteries, while the second tier uses the inflow rate determined from the first tier as the coronary inflow boundary condition and then estimates the vascular bed resistances for the patient‐specific coronary arterial network that was extracted from the CCTA image data.

##### 2.2.2.1 First tier of the parameter estimation

The first‐tier of the parameter estimation utilising the closed‐loop cardiovascular model with an initial and adaptive parameter estimation technique that are described in the previous studies.^66^, ^72^ The purpose of this technique is to provide an estimate for the volumetric flow rate at the inlet of the coronary arteries and to provide an estimate for the left and right ventricular pressures, as they add an external pressure to the coronary vascular beds. The first tier is performed twice: once for hyperaemic conditions and once for resting conditions. As no clinical data on patient pressures or heart rates were available, only population averaged values from literature were used. The population averaged values for both hyperaemic and resting conditions (assumed the same for all patients) are described in Table 3 and are from population averages of previous coronary artery studies.^18^, ^19^ The assumption that the values of parameters such as the heart rate and blood pressures are consistent among patients is valid as FFR has been shown to be independent of heart rate, blood pressure, and heart contractility^12^, ^73^; the relationship between iFR and FFR is also independent of heart rate,^26^ and iFR has also been shown to be independent of heart rate, blood pressure, and heart contractility.^25^

**Table 3:** Parameters used in the model for resting and hyperaemic conditions

	Resting	Hyperaemic
Systolic pressure, mmHg	115	115
Diastolic pressure, mmHg	74	70
Cardiac output, L/min	5.19	7.6
Heart rate, BPM	65	90

An additional change from the resting condition to the hyperaemic condition is performed by reducing the total coronary vascular bed resistance by 78*%*, this causes the mean flow rate in the coronary arteries for the hyperaemic condition to be 3.5 times larger than in resting conditions.^18^ This means that there are only two different defined inflow waveforms for the coronary arteries, which allow a more straightforward comparison between cFFR and cIFR.

##### 2.2.2.2 Second tier of the parameter estimation

The inlet boundary condition for all simulations is a defined flow rate (one for hyperaemic conditions and one for resting conditions) that was generated by the closed‐loop cardiovascular model described in the first parameter estimation tier. The second tier uses the patient‐specific coronary geometry that was extracted via segmentation. The outlet boundary condition is a lumped‐parameter model that includes external pressure from the left and right ventricles^66^ (saved from the first tier of the parameter estimation). The total coronary resistance of each branch is calculated using the commonly used relation:

(7) $$R_{i}=\frac{\frac{1}{3}\text{Systolic Pressure}+\frac{2}{3}\text{Diastolic Pressure}}{Q_{i}},$$

where the subscript *i* represents the branch (left or right) of the coronary artery network, and *Q*_*i*_ is the defined inflow into the coronary branch from the first tier. The resistance of each branch is then distributed to each terminal vessel using the proximal Murray's law with a power of 2.27.^74^ This means that the second tier of parameter identification relies solely on the patient specific geometry for the distribution of the vascular resistance.

##### 2.2.2.3 Model cFFR and ciFR

**Figure 1:**
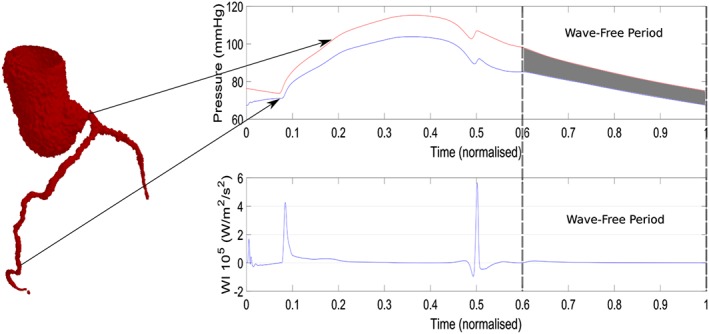
Overview of the instantaneous wave‐free ratio, iFR (the average of the ratio *P*_*d*_/*P*_*p*_ in the shaded region) and an example of the wave‐free period during diastole

Both conventional FFR and iFR are measurements that involve the ratio of the pressure proximal (*P*_*p*_) to a stenosis (usually aortic pressure) and the pressure distal (*P*_*d*_) to a stenosis. The ratio of *P*_*d*_/*P*_*p*_ is used for all cases. The main differences between these two methods are as follows: conventional FFR is measured under maximal hyperaemic conditions, which requires a drug such as adenosine to be administered and is the mean of the pressure ratio *P*_*d*_/*P*_*p*_ over one cardiac cycle (in clinical practice multiple cycles); iFR is performed under resting conditions and is the mean of the pressure ratio *P*_*d*_/*P*_*p*_ during the wave‐free period, which is shown in Figure 1 as the grey shaded region. In this model, ciFR is assumed to begin 1/5*th* into diastole to the end of the cardiac cycle.

## 3 RESULTS

### 3.1 cFFR comparison with clinical invasive FFR

**Figure 2:**
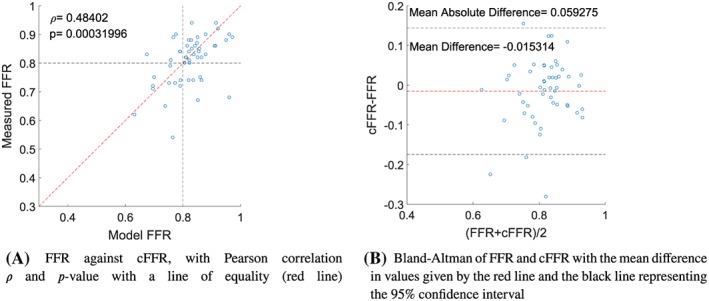
Comparison of cFFR with the invasive FFR measurements. cFFR, computed fractional flow reserve

An overview of results for the reduced‐order cFFR model in comparison with the invasive clinical FFR measurement is shown in Table 4 with the correlation and Bland‐Altman graphs shown in Figure 2. The diagnostic accuracy of the model is 75.76*%*. The mean absolute difference between the cFFR and FFR values is 0.059, with a mean difference of −0.015 and a standard deviation of 0.0813. A Pearson coefficient of 0.484 shows a moderate linear correlation between the cFFR and invasive FFR values.

**Table 4:** Diagnostic results of cFFR prediction showing the total number of true positive, false positive, true negative, false negative, sensitivity, specificity, positive predictive value, negative predictive value, and diagnostic accuracy

No. of true positive	15
No. of false positive	10
No. of true negative	35
No. of false negative	6
Sensitivity, *%*	71.43
Specificity, *%*	77.78
PPV, *%*	60.00
NPV, *%*	85.37
Diagnostic accuracy *%*	75.76

Abbreviations: cFFR, computed fractional flow reserve; NPV, negative predictive value; PPV, positive predictive value.

### 3.2 Correlation between cFFR and ciFR

**Figure 3:**
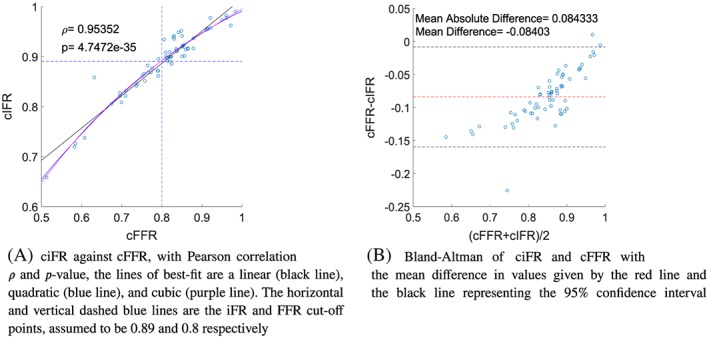
Comparison of the model predicted cFFR and ciFR values. cFFR, computed fractional flow reserve; ciFR, computed instantaneous wave‐free ratio

The main results are shown in Figure 3 with a Pearson correlation coefficient of *ρ*=0.95352 showing a strong linear correlation between cFFR and ciFR values. The Bland‐Altman plot shown in Figure 3B shows the mean difference between cFFR and ciFR to be 0.08402.

Lines of best‐fit can be used to aid determining the correct relationship between cFFR and ciFR, as many correlation measures are based on the assumption of a linear relation. Figure 3A compares ciFR to cFFR using the the following polynomials of best‐fit: the linear polynomial

(8) $${\text{ciFR}}_{1}=0.6469\text{cFFR}+0.3688,$$

the quadratic polynomial

(9) $${\text{ciFR}}_{2}=-0.5926{\text{cFFR}}^{2}+1.5616\text{cFFR}+0.0218,$$

and the cubic polynomial

(10) $${\text{ciFR}}_{3}=0.6770{\text{cFFR}}^{3}-2.1472{\text{cFFR}}^{2}+2.7307\text{cFFR}-0.2654.$$

The polynomials of best fit allow the determination of the cut‐off point for iFR by comparing the iFR value to the diagnostic cut‐off point of FFR=0.8. The linear polynomial estimates a value of ciFR_1_=0.8863, the quadratic polynomial estimates ciFR_2_=0.8918, and the cubic polynomial estimates ciFR_3_=0.8916. The results indicate that the relationship between FFR and iFR is better described by a second‐order polynomial that provides an excellent fit for the data.

#### 3.2.0.4 Receiver operating characteristic curve

**Figure 4:**
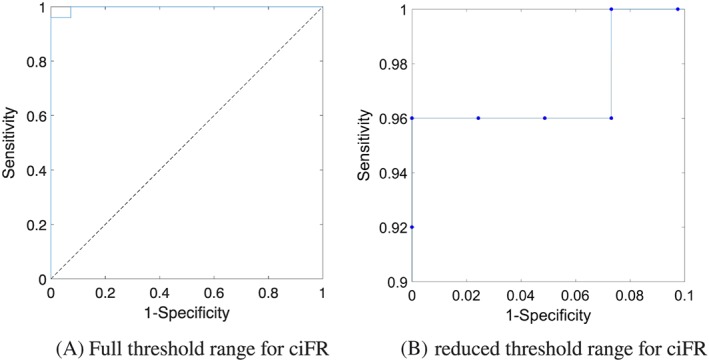
Receiver operating characteristic curve of cFFR and ciFR over the a range of threshold points for ciFR. cFFR, computed fractional flow reserve; ciFR, computed instantaneous wave‐free ratio

It is common to use receiver operating characteristic curves (ROC) to determine the optimum threshold of a methodology. Figure 4 shows the ROC curve of ciFR for different threshold values, when compared with cFFR (with a threshold of 0.8), where the area under the curve is AUC=0.9971. The optimum threshold for ciFR via ROC analysis is determined to be ciFR=0.8910.

## 4 DISCUSSION

The performance of the reduced‐order cFFR methodology in comparison with the invasive FFR measurements is very satisfactory at this early stage of development and shows a similar level of accuracy to several three‐dimensional cFFR methodologies.^75^ The diagnostic accuracy of the reduced‐order methodology is 75.76*%*, which is in the region seen from other studies; however, the current methodology did not have patient information along with the CCTA data, and hence, a population average blood pressure, heart rate, and cardiac output from published studies^18^, ^19^ was chosen. The utilisation of routinely measured patient data may improve the models estimation of cFFR by integrating more patient‐specific parameters. Even with the lack of patient‐specific data the strategy performed well with a sensitivity of 71.43*%*, a specificity of 77.78*%*, a positive predictive value of 60*%* and a negative predictive value of 85.37*%*. Furthermore, the mean difference between cFFR and invasive FFR was −0.015314, which is lower in magnitude than the mean difference shown in the three‐dimensional methodology performed for different patient data in the DISCOVER‐FLOW^43^ (0.022), DeFACTO^44^ (0.058), and NXT^45^ (0.03) studies. The standard deviation of the reduced‐order model is 0.0813, which is close to the lowest standard deviation from the other studies of 0.074.^45^ The model‐predicted cFFR showed a lower Pearson correlation with the FFR than in other studies^75^; however, this could be attributed to the smaller cohort size and the low range of measured FFR values, as the majority (78.43*%*) of invasive cFFR measurements were in the range 0.7< FFR <0.9. Overall, the results indicate that the reduced‐order methodology presented here provides a high level of diagnostic accuracy for cFFR.

The framework presented does need to be tested on a larger cohort in fully blinded conditions and ideally using prospective data. In the current cohort, there are several patients that would have been excluded in other studies because of poor image quality^75^; however, the diagnostic performance of the methodology was not the only objective of this paper. The main reason for the comparison at this stage was to validate the methodology and provide confidence that the predicted cFFR values of the model are close to the invasive FFR measurements, which in turn will provide an indication that the iFR values predicted by this model can be trusted. The methodology also allows the computed iFR values to be implemented within the same framework as cFFR, and thus, the developed software could easily be adapted to automatically perform the hybrid iFR‐FFR technique.^38^, ^40^ The use of the same inlet volumetric flow rate and estimated heart rate also ensures that only the geometry and downstream resistance of the coronary network are the main variables that affect the FFR and iFR value predictions.

### 4.1 cFFR and ciFR comparison

The use of iFR in combination with or instead of FFR has received increased attention in recent years. However, many studies use different threshold values of iFR to determine whether a patient needs further treatment.^36^ The estimation of the iFR diagnostic threshold ranges from 0.92,^76^ 0.9,^29^, ^31^, ^77^, ^78^ 0.89,^79^, ^80^ 0.88,^81^ and even 0.83,^82^ which obviously indicates that the best diagnostic threshold for iFR is still not known. The results from this paper indicate that the diagnostic threshold for iFR is close to 0.89 and that the relationship between ciFR and cFFR is quadratic in nature. This agrees with the observed behaviour from a comparison of invasive FFR and iFR measurements.^39^ Other studies have reported an approximately linear behaviour from measurements^32^, ^83^ and from a Monte Carlo simulation with a lumped parameter model^64^; however, there is a noticeable deviation from the linear line of best fit at lower FFR and iFR values, which indicates that a nonlinear relationship would provide a better fit between FFR and iFR measures.

**Figure 5:**
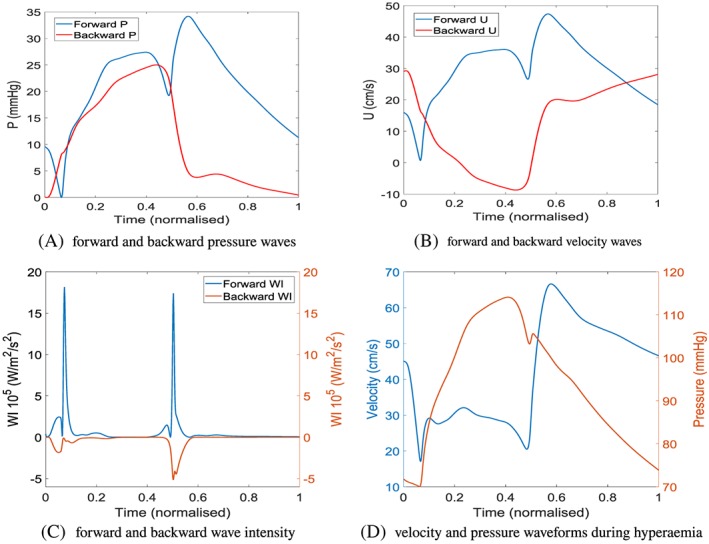
Separation of forward and backward‐propagating waves in the left main coronary artery

Another interesting aspect to consider is whether the cardiovascular system is a pressure‐driven or flow‐driven system, which depends on whether the heart is a flow generator or pressure generator.^84^ The fact that the heart contracts and creates a pressure that acts on coronary capillary vessels complicates matters as there would be a forward‐propagating pressure wave originating from the heart that travels into the aorta and then through the coronary arteries from the proximal to distal location and also a backward‐propagating pressure wave that originates from the coronary capillaries as a result of the contracting heart that travels from the distal region of the coronary arteries to the proximal region.^85^ In reality ,it is more likely that the cardiovascular system is a complex combination of both pressure‐driven and flow‐driven phenomena.^84^ However, because of the forward and backward pressure waves during systole and at the start of diastole, which can be seen in Figure 5, a pressure index based on the diastolic phase could be more reliable as the lowest resistance to flow in the coronary arterial network is observed during diastole.

Many of the fundamental assumptions needed for FFR have since been proven incorrect. For example, the pressure and flow are assumed to be directly proportional (have a linear relationship) when the resistance is constant and minimal^86^ but has since been shown have a curvilinear relationship experimentally^87^ and mathematically. This essentially means that FFR relies on the assumption that the characteristics of flow are the same in both diseased and healthy vessels. The assumption of the microcirculatory resistance being constant and minimal during hyperaemia is also questionable and has been proven incorrect in the presence of several conditions including microvascular dysfunction.^86^ In hyperaemia, the resistance averaged over a cardiac period may be consistent during hyperaemia, but the microvascular resistance will vary significantly over a cardiac cycle as the heart contraction squeezes on the microcirculation during systole that increases resistance.^88^ These assumptions are not required for iFR; instead, the main assumption for iFR is that the resistance of the microcirculation is stable during the wave‐free period, which is during a period in diastole where no waves are generated, such as seen in Figure 5C from a normalised time of 0.6 to 1.

Many studies compare the diagnostic performance of iFR to FFR and consider the latter index to be the “perfect measure.” This generally means that iFR looks worse than FFR, but this is a rather unfair comparison. Further studies have indicated that iFR and FFR have a similar number of negative cardiovascular events after 1 year^41^ and thus is at least on‐par with FFR. Several studies have compared iFR and FFR^29^, ^30^, ^31^, ^32^, ^33^, ^34^, ^35^, ^36^, ^37^; however, only one attempted to explain the cause of the various diagnostic disagreements observed between iFR and FFR.^39^ The study concluded that it was actually the FFR that was likely overestimating the severity of the stenosis because of the hyperaemic condition rather than iFR underestimating the stenosis, and for the cases that had a positive FFR but negative iFR, the observed coronary flow characteristics were similar to that seen in angiographically unobstructed vessels, which indicates that iFR may be the more reliable and suitable measure.

From a non‐invasive standpoint for the determination of iFR and FFR, iFR has more advantages over FFR as it does not require an estimation of how the patient will react to the administered hyperaemic inducing drug. Many cardiac and haemodynamic parameters can be measured by non‐invasive means, including cardiac output estimations, brachial artery blood pressures, and heart rate. These parameters can be directly utilised by any non‐invasive iFR model predictions rather than attempting to predict the effects of a hyperaemic condition, which are variable between patients and are required for FFR estimations. In addition, in the majority, the patient CCTA scans are performed at resting conditions. This is particularly important as the inducing of hyperaemic conditions through the use of a drug, such as adenosine, was observed to increase coronary vessel diameters by up to 15%^89^; thus, there is even significant uncertainty for cFFR regarding the actual patient geometry that is extracted from CCTA data.

### 4.2 Limitations

The main limitations of this study are that the size of the cohort is relatively small with 52 patients and a total of 66 stenoses. Furthermore, we assume that there are no pressure losses at vessel junctions and do not have patient data such as age, blood pressures, heart rate, or gender, which may play a role in FFR prediction. We do not have any invasive iFR measurements; only FFR measurements and the patient CCTA images were available. As the data are retrospective, we do not know the exact location of the pressure measurement taken during invasive FFR, as only the general location is normally recorded in the clinic. However, the main target of this study was to determine the diagnostic threshold of iFR in a more controlled environment, and although the cFFR performance of the model is very good, it was not the focus of this paper, and thus, the additional patient data are not of importance here.

It has been observed that vessels with coronary artery disease^90^ can cause a long‐term auto‐regulatory response in the coronary micro‐vasculature resistance to preserve flow. This may impact the accuracy of the flow estimates. However, it is not known how or if this auto‐regulation affects flow rates in hyperaemia and would add an additional unknown and source of uncertainty in the model, which will make comparison between FFR and iFR more challenging. Thus, we have not included this auto‐regulatory response in this study.

## 5 CONCLUSIONS

The diagnostic performance of the cFFR with the invasive FFR measurement is very promising. The methodology not only showed a lower correlation between cFFR and FFR than in other studies but also showed the lowest magnitude of mean difference and one of the lowest standard deviations between the computed and measured FFR when compared with other studies. The lower correlation coefficient may be due to the lower patient numbers considered in this cohort and also on the nonselective nature in this study with regard to image quality such as the presence of significant motion artefacts, blooming artefacts, and high levels of calcification that can have a large impact on the accuracy of the segmentation process. The methodology must now be performed on a significantly larger cohort in a fully blinded fashion and ideally prospectively in order to improve the confidence in the 1D modelling methodology for both FFR and iFR predictions.

A comparison between cFFR and computed iFR was also performed by utilising the patient CCTA data. The model predicted an iFR diagnostic cut‐off point of 0.891 with the correlation and polynomial of best fit between cFFR and ciFR being quadratic in nature. Further studies involving comparisons between FFR and iFR must be performed in order to determine whether FFR or iFR is the more reliable measure. There is a significant advantage of iFR, as it does not require a hyperaemic drug infusion which can cause negative side affects in patients and would also be less expensive. When considering a computational methodology for prediction purposes, ciFR is also more attractive as a diagnosis index when compared with cFFR, as the patients non‐invasive measurements can be utilised directly without the need to predict hyperaemic conditions.
